# Application and Development of EEG Acquisition and Feedback Technology: A Review

**DOI:** 10.3390/bios13100930

**Published:** 2023-10-17

**Authors:** Yong Qin, Yanpeng Zhang, Yan Zhang, Sheng Liu, Xiaogang Guo

**Affiliations:** 1 Institute of Advanced Structure Technology, Beijing Institute of Technology, Beijing 100081, China; 3120226049@bit.edu.cn; 2 Beijing Perfect-Protection Technology Co., Ltd., Beijing 101601, China; 13701246897@139.com (Y.Z.); 13601336866@139.com (Y.Z.); 13601131795@139.com (S.L.)

**Keywords:** EEG, acquisition devices, electrodes, classification algorithm

## Abstract

This review focuses on electroencephalogram (EEG) acquisition and feedback technology and its core elements, including the composition and principles of the acquisition devices, a wide range of applications, and commonly used EEG signal classification algorithms. First, we describe the construction of EEG acquisition and feedback devices encompassing EEG electrodes, signal processing, and control and feedback systems, which collaborate to measure faint EEG signals from the scalp, convert them into interpretable data, and accomplish practical applications using control feedback systems. Subsequently, we examine the diverse applications of EEG acquisition and feedback across various domains. In the medical field, EEG signals are employed for epilepsy diagnosis, brain injury monitoring, and sleep disorder research. EEG acquisition has revealed associations between brain functionality, cognition, and emotions, providing essential insights for psychologists and neuroscientists. Brain–computer interface technology utilizes EEG signals for human–computer interaction, driving innovation in the medical, engineering, and rehabilitation domains. Finally, we introduce commonly used EEG signal classification algorithms. These classification tasks can identify different cognitive states, emotional states, brain disorders, and brain–computer interface control and promote further development and application of EEG technology. In conclusion, EEG acquisition technology can deepen the understanding of EEG signals while simultaneously promoting developments across multiple domains, such as medicine, science, and engineering.

## 1. Introduction

The brain is the most advanced part of the nervous system and possesses immensely powerful functions and complex structures. Electroencephalogram (EEG) is a significant bioelectric signal produced by neural activity that reflects the physiological processes of neurons in the brain and contains a wealth of brain activity information [1,2]. The signal decoding of EEG acquisition devices and intelligent device control has become a hot topic in contemporary neuroscience research [3,4].

**Figure 1:**
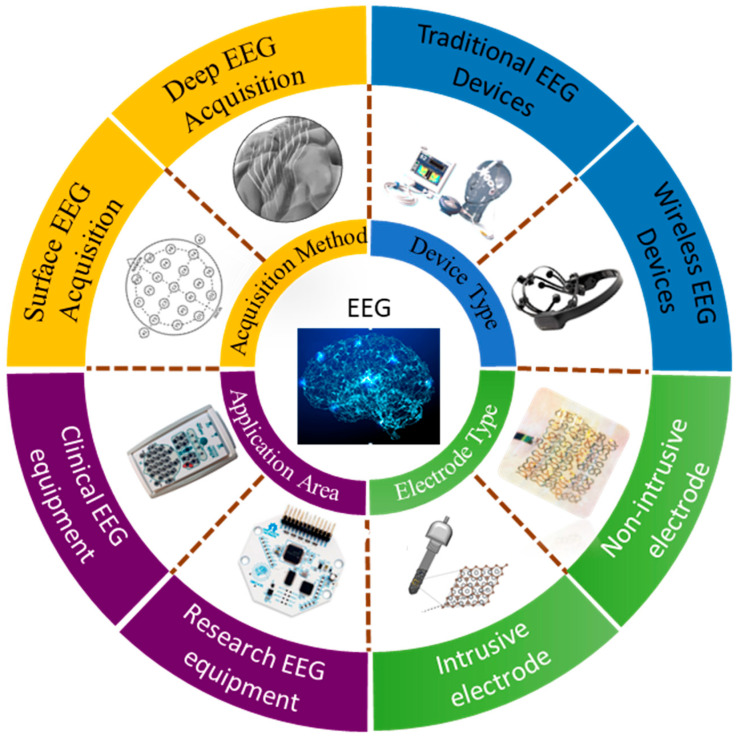
Overview of the classification of EEG acquisition devices from perspectives of acquisition method, device type, electrode type, application area. Reprinted with permission from [5]. Copyright 2016, The Neurodiagnostic Journal. Reprinted with permission from reference [6]. Copyright 2018, ACS Applied Materials & Interfaces. Reprinted with permission from reference [7]. Copyright 2023, https://www.chem17.com/st377869/product_30998972.html (accessed on 29 August 2023). Reprinted with permission from [8]. Copyright 2023, https://www.emotiv.com/epoc-x (accessed on 29 August 2023). Reprinted with permission from [9]. Copyright 2023, ACS Applied Electronic Materials. Reprinted with permission from [10]. Copyright 2022, Nano Letters. Reprinted with permission from [11]. Copyright 2023, https://openbci.com (accessed on 29 August 2023). Reprinted with permission from [12]. Copyright 2023, https://compumedicsneuroscan.com (accessed on 29 August 2023).

Figure 1 shows the four prevalent classification methods pertaining to EEG acquisition devices. These methods include acquisition approach, device type, electrode type, and application domain. The acquisition approaches are categorized into two types: surface [5] and deep [6] EEG. Surface EEG involves the placement of electrodes on the scalp to measure the electrical activity of the brain. Deep EEG refers to the use of intracranial electrodes implanted within brain tissue to record brain signals. Classification by device type includes traditional and wireless EEG devices. Traditional EEG devices employ conventional electrode placements and require wired connections between the electrodes and amplifiers or recording systems [7]. Wireless EEG devices utilize wireless transmission technology, eliminating the need for wired connections and offering greater flexibility and convenience [8]. Regarding electrode type, the two categories are invasive and noninvasive electrodes. Noninvasive electrodes collect EEG signals through the scalp, offering a relatively safe and surgery-free approach, thus having broader applications. Invasive electrodes typically provide higher spatial resolution, enabling more accurate and fine-grained EEG signal acquisition; however, they require surgical implantation, making them riskier [9,10]. Finally, the application domain of EEG devices includes research-grade and clinical EEG devices. Research-grade devices are employed for scientific investigations and feature higher sampling rates, more channels, and increased precision. Clinical EEG devices conform to clinical standards and are used for medical diagnosis, monitoring, and treatment [11,12].

EEG acquisition and feedback devices can transform brainwave signals acquired from the brain into command signals that can be understood by computers or other external devices [13], enabling direct interaction between humans and machines. This empowers individuals to control computers and external devices through conscious thought, enhancing the efficiency of tasks at work and in daily life.

With the advancement of neuroscience, computer technology, and signal processing, EEG acquisition technology has undergone extensive research and application over the past few decades [14,15]. For instance, the Neuroscan EEG system is the most advanced and widely used in the industry One of the EEG acquisition devices [12]. Additionally, Electrical Geodesics (EGI) Inc. (Eugene, OR, USA), founded in 1992, has gained recognition for its flagship product, GES400, which boasts two key features: compatibility with magnetic resonance imaging (MRI) and the use of mesh electrodes [16]. Another noteworthy EEG acquisition system is the NeuSen W, introduced by Changzhou Bori Kang Technology Co., Ltd. (Changzhou, China). This wireless EEG collection device has been specifically designed for applications in brain–computer interfaces (BCI) [17]. Moreover, in some universities and research institutions, research and applications in the field of EEG systems have become more profound and specialized. For example, under the leadership of Zhang Wenchang, a research team at Tsinghua University has successfully integrated automatic robot control technology with brain–computer interface (BCI) technology. This integration has led to the development of an asynchronous BCI-shared control system based on motor imagery. In experimental trials, participants were able to control a robotic arm for navigating around obstacles and manipulating objects solely through motor imagery, achieving an impressive accuracy rate of 80% [18].

Generally, research on EEG acquisition and feedback technology has reached a relatively mature stage, with various research institutions and companies applying it extensively in the medical, military, entertainment, and other fields. There are many comprehensive research studies related to EEG acquisition and feedback devices. For instance, J. Wang et al. have explored the latest classifications and future development directions of flexible electrodes to facilitate the advancement of EEG electrode technology [19]. In the realm of EEG classification algorithms, L. R. Quitadamo et al. have conducted a review on support vector machine detection methods for human–machine interaction physiological models based on EEG and EMG (electromyography) signals. EEG classification algorithm reviews contribute significantly to researchers’ understanding of the current status of EEG classification algorithms, optimization of research designs, enhancement of algorithm performance, and addressing challenges within the field [20]. After conducting the relevant research, it has been identified that there is a relative scarcity of comprehensive reviews on the entire EEG acquisition and feedback system. Therefore, this paper provides a comprehensive overview of the application and development of EEG technology, focusing on aspects such as EEG signal acquisition and processing, applications of EEG technology, and the utilization of EEG classification algorithms. Furthermore, the existing challenges and technological bottlenecks in EEG technology are discussed, followed by a summary of the trends and directions for the future development of EEG technology.

The following article will systematically investigate the application and development of EEG acquisition technology in four main sections: “Section 2”, “Section 3”, “Section 4” and “Section 5”.

## 2. The Acquisition and Processing of EEG Acquisition and Feedback Devices

### 2.1. The Acquisition and Feedback Principle of EEG Acquisition Devices

**Figure 2:**
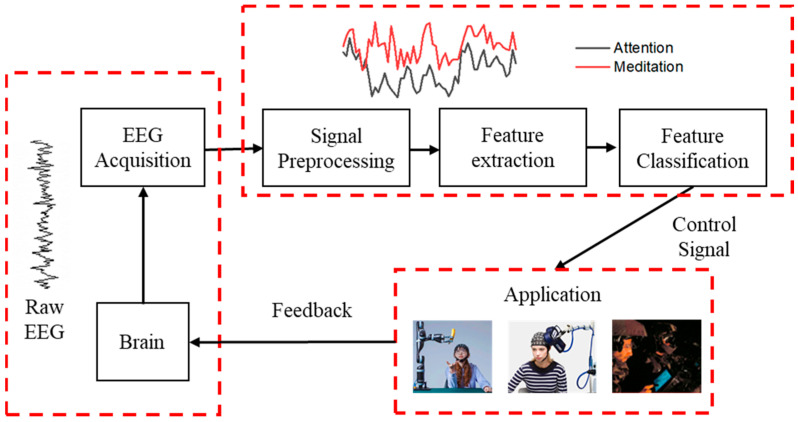
The overall framework of an EEG real-time acquisition and monitoring system.

EEG acquisition devices serve as a data source for various EEG applications and studies. As a precision testing instrument, it must ensure user safety while maintaining high accuracy and a high common-mode rejection ratio (CMRR) of the system. Additionally, efforts have been made to minimize the power consumption, size, and cost of the system [1,21]. A complete EEG acquisition and feedback system typically comprises three main parts: EEG signal acquisition, EEG signal processing, and control feedback, as shown in Figure 2. The acquisition system first collects EEG signals using an EEG acquisition device. After preprocessing steps such as artifact removal, feature extraction is performed, and once the feature vector of the subject is obtained, it undergoes classification and recognition. Finally, the recognized result is converted into corresponding control/feedback instructions for the output [22].

### 2.2. Composition of EEG Signal Acquisition and Feedback Devices

The components of the EEG acquisition system include EEG electrodes, preprocessing circuits, analog-to-digital converters (ADC), and EEG signal control and feedback [23,24,25,26]. The EEG acquisition system must ensure high accuracy and low-noise measurements while prioritizing user safety and comfort [27,28]. Each component plays a critical role in the system. The following section introduces the types of EEG electrodes used and provides an overview of each type.

#### 2.2.1. EEG Acquisition Electrode Type

**Figure 3:**
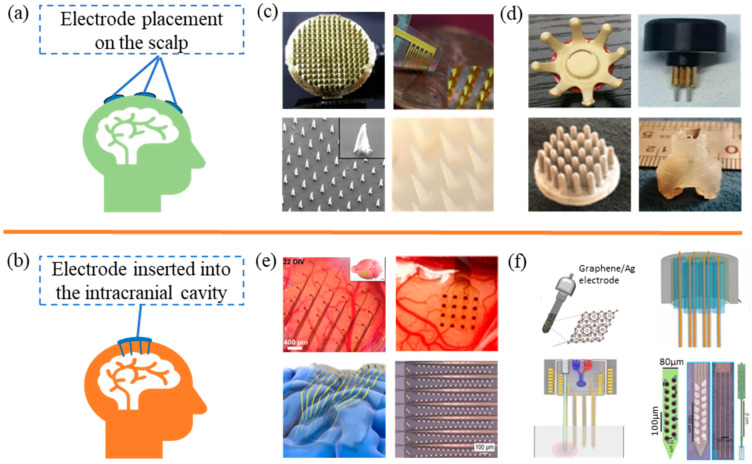
Electrode types. (**a**) Non-invasive EEG electrodes. (**b**) Invasive EEG electrodes. (**c**) Micro-needle dry electrode. Reprinted with permission from reference [32]. Copyright 2012, Sensors and Actuators A: Physical. Reprinted with permission from reference [33]. Copyright 2017, Sensors and Actuators B: Chemical. Reprinted with permission from reference [34]. Copyright 2015, Sensors and Actuators A: Physical. Reprinted with permission from reference [35]. Copyright 2021, ACS Applied Nano Materials. (**d**) Finger-type dry electrode. Reprinted with permission from reference [36]. Copyright 2018, Scientific reports. Reprinted with permission from reference [37]. Copyright 2020, Scientific reports. Reprinted with permission from reference [38]. Copyright 2018, Scientific Reports. Reprinted with permission from reference [39]. Copyright 2021, Sensors and Actuators A: Physical. (**e**) Thin-film invasive EEG electrodes. Re-printed with permission from reference [40]. Copyright 2022, Advanced Functional Materials. Re-printed with permission from reference [41]. Copyright 2019, Nano Letters. Re-printed with permission from reference [6]. Copyright 2018, ACS Applied Materials and Interfaces. Reprinted with permission from reference [42]. Copyright 2019, Journal of Medical Internet Research. (**f**) Straight-beam invasive EEG electrodes. Reprinted with permission from reference [10]. Copyright 2022, Nano Letters. Reprinted with permission from reference [43]. Copyright 2020, Science Advances. Reprinted with permission from reference [44]. Copyright 2019, Nature Communications. Reprinted with permission from reference [45]. Copyright 2019, Neuron.

In the fields of medicine, neuroscience, psychology, and various brain signal research domains, Ag/AgCl electrodes are widely regarded as one of the most commonly used EEG electrodes due to their excellent signal-to-noise ratio, reliability, stable signal quality, and cost-effectiveness. They serve as the standard against which the performance of other electrode types is often compared. However, Ag/AgCl electrodes require the use of conductive gel, and the electrode preparation and application processes can be complex and time-consuming [29]. Therefore, to meet the diverse requirements of different application areas for EEG electrodes, there has been a proliferation of various types of EEG electrodes. These electrodes have emerged as alternatives to address the challenges associated with Ag/AgCl electrodes while catering to specific research needs. There are various classification methods for EEG acquisition electrodes. They can be divided into two types according to their placement on the brain: non-invasive and invasive [30,31], as shown in Figure 3a,b. Non-invasive uses electrodes placed on the scalp to collect EEG signals and achieves human–machine interaction through signal processing techniques. Invasive involves implanting electrodes into the cerebral cortex to obtain more accurate signals; however, it requires surgical procedures and carries higher risks.

As shown in Figure 3c,d, micro-needle and finger-like non-invasive electrodes are two typical non-invasive EEG electrodes. Micro-needle non-invasive EEG electrodes represent an innovative brainwave electrode technology designed to achieve higher-quality EEG signal acquisition without compromising scalp integrity. Conventional EEG electrodes typically require adhesive gels or conductive pastes to attach to the scalp, which can lead to discomfort and skin sensitivity. Contrastingly, micro-needle non-invasive EEG electrodes employ micro-fine needle-like electrode tips that gently puncture the scalp, allowing for closer proximity to neurons, thereby enhancing signal quality [32,33,34,35]. Compared to traditional wet electrodes, finger-like EEG electrodes not only eliminate the discomfort or skin sensitivity caused by adhesive gels or conductive pastes adhering to the scalp but also easily penetrate hair, thereby enhancing the quality and accuracy of EEG signal acquisition. Additionally, in contrast to needle-type non-invasive electrodes, finger-like EEG electrodes do not puncture the scalp, making them relatively safer and more comfortable [36,37,38,39]. Therefore, both micro-needle and finger-like non-invasive electrodes, through their structural designs, enable direct contact with the scalp, effectively reduce the impedance between the scalp and electrodes, and provide more stable and reliable EEG signals. Additionally, compared with traditional electrodes, these two types of electrodes exert less pressure on the scalp, alleviating discomfort during extended wear. Non-invasive EEG electrodes have potential applications in fields such as neuroscience research, brain–computer interface technology, and cognitive assessment. They offer researchers more accurate EEG signal data, thus facilitating a deeper understanding of brain activity [32,33,34,35,36,37,38,39].

Compared to invasive brain EEG electrodes, the fabrication process for micro-needle and finger-like non-invasive EEG electrodes is relatively straightforward. For instance, using 3D printing technology, the electrode models are initially designed with 3D modeling software. Subsequently, the generated files are loaded into a 3D printer, which uses an insulating flexible polymer to create the electrode’s structure. Finally, a layer of conductive metal is deposited on the flexible electrode surface to complete the EEG electrode production [32]. Using photolithography techniques involves coating a clean substrate with photosensitive photoresist, exposing the photoresist to define patterns, developing to remove unwanted portions, and going through cleaning and baking steps to form the desired microstructures. Finally, conductive metal is either coated or sputtered onto the microstructure [33,34,35]. Utilizing molding techniques, which is a common fabrication method, involves steps such as preparing raw materials, designing, and manufacturing molds, heating, loading, applying high pressure, curing through heating, or cooling, opening the mold, trimming, and inspecting. Once the flexible finger-like structure is formed, a layer of conductive metal is applied to its surface to create the EEG electrode [36,39].

As shown in Figure 3e,f, thin-film invasive EEG electrodes and straight-beam invasive EEG electrodes are two typical invasive EEG electrodes. A thin-film invasive brain electrode is a specific type of invasive electrode that employs relatively thin materials, often in the form of flexible films, for monitoring and recording brain neural activities. These electrodes are designed to be more flexible in shape and structure, allowing them to adapt better to the curves and topology of the brain surface [6,40,41,42]. Columnar invasive brain electrodes resemble slender columns or needles. These electrodes are surgically implanted into the brain tissue to capture the electrical activities of neurons. Their design aims to provide more precise positional tracking and a higher spatiotemporal resolution, enabling researchers to observe and analyze neural network activities in greater detail [10,43,44,45]. Furthermore, invasive brain electrodes capture neural electrical activity by inserting the electrodes into the interior of the brain tissue. These can be employed for more detailed recordings of specific brain regions, facilitating a deeper understanding of the interactions between neural circuits and functional areas. In clinical settings, they find applications in epilepsy surgery localization and monitoring, as well as in the research and treatment of other neurological disorders. The manufacturing and implantation of invasive brain electrodes present certain challenges, requiring advanced manufacturing techniques to ensure their stability and reliability. Moreover, the implantation process requires highly skilled technical operations and precise positioning to avoid damaging brain tissue. Nonetheless, invasive brain electrodes, including thin-film and columnar variations, play a pivotal role in providing key insights into neural activity and interactions by offering detailed recordings and higher resolution. Thus, it is essential that the complexities of their production and implantation a carefully addressed for successful integration into both research and clinical applications [40,41,42,43,44,46].

For invasive EEG electrodes, due to the need to ensure both signal quality and the safety of electrode penetration into the human body, the electrode fabrication process has higher requirements. The production of thin-film invasive EEG electrodes mainly involves preparing a flexible substrate (such as polyester film like PET or polyimide film like PI), coating with conductive materials (Ag/AgCl or nanocarbon materials like carbon nanotubes or graphene), drying, electrode patterning, adding additional layers, encapsulation, connection and wiring, testing, calibration, and finally packaging [40,41,42]. The fabrication of straight-beam invasive EEG electrodes begins with processing the conductive material (such as gold, silver, stainless steel, etc.) into the desired electrode shape, typically a slender cylindrical form for penetration into brain tissue. An insulating material is then coated onto the surface of the conductive material to ensure electrical conduction only at specific locations. Next, tip design is carried out to ensure accurate electrode penetration into brain tissue, requiring a sharp electrode tip design. Finally, encapsulation and protection are performed to ensure the electrode’s safety and durability [44,45]. Additionally, in some studies, researchers apply materials like graphene to the conductive metal to enhance electrode conductivity, thereby improving the quality of EEG signal acquisition [10].

In conclusion, brain electrodes play a crucial role in EEG data acquisition systems, significantly influencing the quality of recorded brain signals. Therefore, the development of comfortable and convenient wearable brain electrodes that can establish effective contact with the skin or organ tissues is a pressing issue. With the advancement of various flexible fabrication techniques, such as the assembly of complex 3D structures, it is anticipated that the current challenges associated with brain electrodes will be effectively addressed [47,48,49,50].

#### 2.2.2. Analog-to-Digital Conversion Circuit

In EEG signal acquisition, the ADC is the core component responsible for converting analog voltage signals into digital form. The ADC performance significantly affects the quality and accuracy of EEG signals [51].

First, ADC resolution is a crucial factor affecting the EEG signal acquisition quality [52]. A higher resolution allows the ADC to convert smaller voltage variations, thus improving signal accuracy. The ADC sampling rate is another key parameter [53]. The sampling rate determines the number of data points that the ADC acquires per second, and an appropriate sampling rate must be selected for different types of EEG experiments to satisfy the experimental requirements. Second, the noise level of the ADC is also a vital factor influencing signal quality. Quantization and circuit noise in the ADC can reduce the signal-to-noise ratio of the EEG signals [54]. Hence, low-noise ADCs must be employed, and noise suppression techniques, such as differential output and filtering, should be incorporated during the design process to enhance the signal quality. Furthermore, the effect of power-supply noise must be considered for the ADC [55]. Since ADCs are sensitive to power-supply noise; stable power supplies are required to minimize their influence on signals. Moreover, the selection and positioning of the reference electrodes must be considered to avoid the introduction of power-supply noise. Finally, factors such as power consumption and size should also be considered for the ADC [56]. An ADC with a lower power consumption is more reliable for prolonged data acquisition experiments, whereas a smaller ADC is more suitable for portable devices.

**Figure 4:**
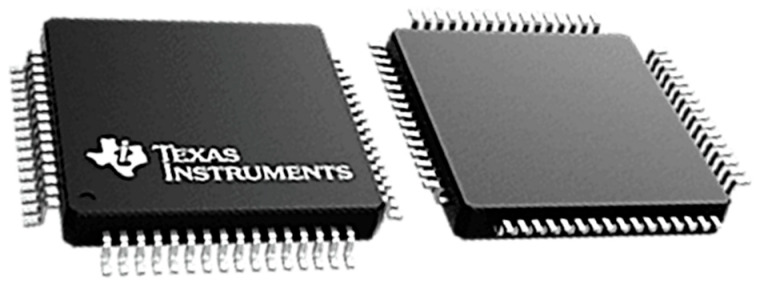
The ADS1299 series acquisition chip.

In summary, ADC plays a crucial role in EEG acquisition devices. To obtain high-quality EEG signals, factors such as ADC resolution, sampling rate, noise, power-supply noise, reference electrodes, power consumption, and size must be considered [57,58,59,60]. As shown in Figure 4, TI’s ADS1299 series is a low-noise 4, 6, and 8-channel, 24-bit ADC chip designed for EEG and biopotential measurements. This series features programmable gain amplifiers (PGA), internal references, and onboard oscillators that provide all the essential features for EEG and ECG applications. With high integration and outstanding performance, ADS1299 allows the construction of scalable medical instrument systems with significantly reduced size, power consumption, and overall cost [61,62]. The ADSD1299 chip launched by XinDong ShenZhou is fully compatible with TI’s ADS1299 and functionally compatible with ADS1298. The chip includes all the commonly used features required for EEG and ECG applications. With its high integration and outstanding performance, ADS1299 enables the creation of various scalable medical instrument systems while significantly reducing their size, power consumption, and total cost [63].

#### 2.2.3. Preprocessing Circuit

During the recording of EEG signals, various noise and artifacts can interfere with the signals, posing significant challenges for signal analysis and processing. Hence, prior to EEG signal recording, preprocessing circuits are typically employed to enhance the signal quality and accuracy by reducing noise and artifacts. Preprocessing circuits generally comprise several main modules, including amplifiers, filters, and reference electrodes. Amplifiers are responsible for increasing the amplitude of EEG signals, making them easier to record [14]. Filters are used to eliminate noise and artifacts from the signals while preserving the main frequency components of the EEG signals. Reference electrodes are typically placed at specific locations on the scalp, away from the primary sources of brain EEG signals. As a result, the potential of the reference electrode is considered as zero or a known potential, used for calculating the potential differences at other electrodes [64,65].

The design of preprocessing circuits must consider multiple factors, such as the signal frequency range, amplification gain, and reference electrode type [66,67,68]. Different types of EEG experiments may require the use of distinct preprocessing circuits to fulfill specific experimental requirements. For instance, Lin et al. [69] designed an analog amplification circuit with a high relevance. The circuit employed an INA128 instrumentation amplifier as a preamplifier, magnifying weak EEG signals by a factor of 1000, and exhibited high input impedance and CMRR. Moreover, they used TL084 as a post-amplifier with a zero-offset circuit to adjust weak EEG signals to a range of 0–5 V. They further applied an analog low-pass filter to anti-alias EEG signals.

In conclusion, the preprocessing circuits play a crucial role in EEG signal acquisition. They reduce interference from noise and artifacts, enhancing signal quality and accuracy. The design of preprocessing circuits should be flexibly chosen according to specific requirements to meet the diverse needs of different types of EEG experiments.

#### 2.2.4. Processor Circuit

In an EEG system, the processor circuit is a core component that enables real-time processing and analysis of raw signals obtained from the EEG acquisition device, laying the foundation for subsequent data-processing and research. Commonly used processor circuits include a field-programmable gate array (FPGA), digital signal processors (DSP), and central processing unit (CPU) [70].

An FPGA processor circuit is preferred because of its high speed, flexibility, and reconfigurability. It can execute various digital signal processing tasks at the hardware circuit level, including digital filtering, spectral analysis, waveform analysis, and time-frequency analysis [71]. Additionally, the FPGA can implement parallel computations for various algorithms, thus improving processing efficiency. The DSP circuit is a specialized microprocessor used for digital signal processing. Compared with traditional general-purpose microprocessors, they offer higher computation speeds and stronger real-time processing capabilities. DSP is commonly employed for implementing signal processing algorithms such as digital filtering, power spectrum estimation, frequency analysis, and time-frequency analysis [72]. Currently, manufacturers such as TI, ADI, and NXP have introduced various DSP chips for EEG signal processing. CPU processor circuits can also be used for EEG signal processing. Compared to FPGA and DSP, CPUs have a slower processing speed, but they possess greater versatility and larger storage capacity. Hence, CPUs are typically used to implement complex algorithms and data-processing tasks such as neural networks and machine learning [73,74]. For example, the HiCCE-128 EEG acquisition system designed by Mannatunga et al. [75] supports EEG decoding by running EEG processing programs on a front-end processor. Similarly, the multimedia control system designed by Shyu et al. [76] utilizes an FPGA chip as the processor to achieve EEG acquisition and decoding functions, enhancing the system’s portability. Praženica et al. [77] compared the performance and cost of DSP and FPGA implementations for ECG signal processing in their research and recommended selecting the appropriate processor for different application scenarios.

In conclusion, the processor circuit plays a crucial role in EEG signal acquisition and processing. Different types of processor circuits have their own advantages and limitations, and their selection should be based on actual requirements and application scenarios.

#### 2.2.5. EEG Signal Control and Feedback

After acquiring the EEG signals, online preprocessing is performed, including eyeblink artifact removal, feature extraction, and pattern classification. Subsequently, the classification results are sent via serial communication to a motion control module based on the DSP TMS32 chip. This module is responsible for controlling specific mechanisms to initiate motion and achieve real-time control of the mechanism through EEG signals. Finally, feedback from the participant, such as pressure, vibration, and temperature signals, is utilized to assess the motion structure status, enabling closed-loop control [78,79,80,81]. This process is depicted in Figure 2, which illustrates a flowchart of the control feedback system.

The integration of EEG acquisition systems with control feedback systems has immense potential in various applications. This technology can be useful in the medical field, such as offering EEG-based biofeedback therapy to individuals with impaired self-regulation and treating neurological disorders [82]. Moreover, it can be applied to control intelligent devices, such as smart helmets. For example, when wearers experience fatigue, distraction, or fainting, a control feedback system can swiftly detect these anomalies and provide timely alerts or prompts, thereby avoiding potential hazards [83,84]. Furthermore, a combination of EEG acquisition systems and control feedback systems can be employed in domains such as smart homes and intelligent transportation, enabling more accurate and convenient control methods [85,86,87,88,89]. In summary, with the continuous advancement of technology, the integration of EEG acquisition and control feedback systems is expected to find widespread applications in an increasing array of domains.

## 3. Application of EEG Acquisition and Feedback System

### 3.1. Emotional Recognition

**Figure 5:**
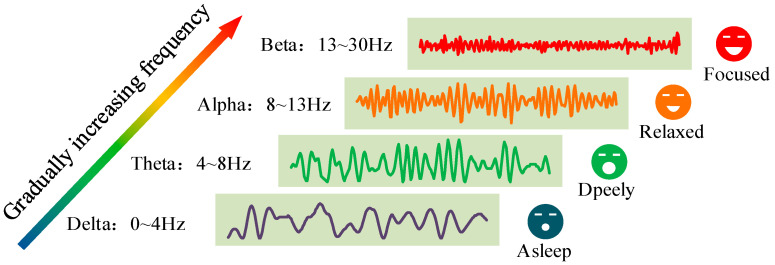
The waveforms of four EEG bands (Delta wave; Theta wave; Alpha wave; Beta wave).

Scientists have classified brainwave signals into different types based on their frequency fluctuations. These waves, in descending order of frequency, are beta, alpha, theta, and delta waves, as shown in Figure 5 [90,91,92,93]. These waves represent the primary components of brainwave activity and can provide insights into various aspects of human consciousness, behavior, and thoughts. For instance, brain wave signals with frequencies ranging from 0 to 4 Hz are referred to as delta waves. They are prominent when individuals are in a deep-sleep state. Brainwave signals in the range of 4 to 8 Hz are known as theta waves. When individuals are in a sleep state, but not deep sleep, theta waves can be detected, indicating a semi-awake state. Brainwave signals in the range of 8–13 Hz are called alpha waves. These waves are observed when the brain is actively engaged in problem solving or deep thinking, reflecting a state of relaxation and inner contemplation. Finally, brainwave signals with frequencies between 13 and 30 Hz are called beta waves. They appear when individuals engage in social interactions, communicate with others, or actively participate in certain daily life activities [94,95,96].

### 3.2. Movement Assistance System

**Figure 6:**
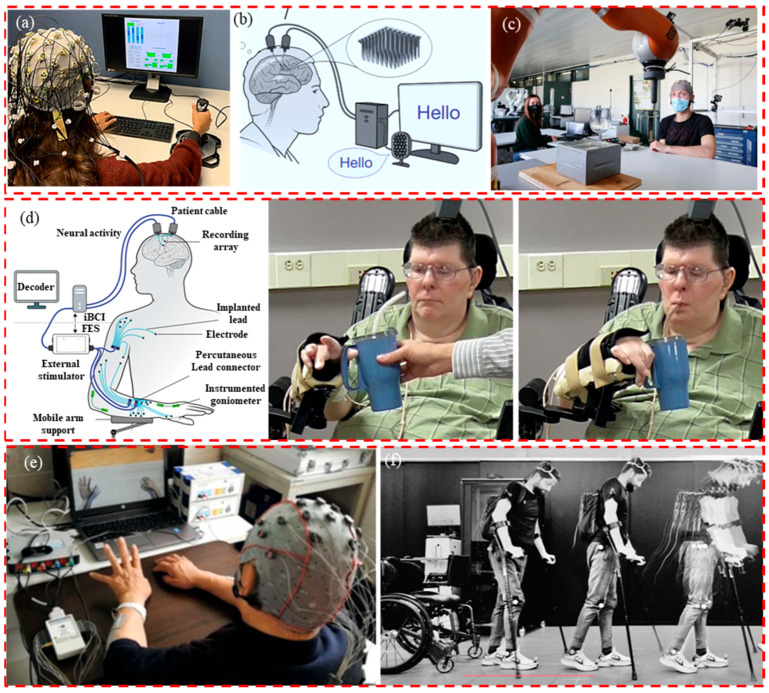
The application of EEG. (**a**) Communication assistance. Reprinted with permission from reference [79]. Copyright 2023, Scientific Data. (**b**) Communication assistance. Reprinted with permission from [97]. Copyright 2023, Nature. (**c**) Robot arm control. Reprinted with permission from [98]. Copyright 2021, Communications Biology. (**d**) Assisted living. Reprinted with permission from [99]. Copyright 2017, The Lancet. (**e**) Medical rehabilitation. Reprinted with permission from [100]. Copyright 2020, Neural Plasticity. (**f**) Medical rehabilitation. Reprinted with permission from [101]. Copyright 2023, Nature.

In the field of movement assistance, EEG data collection plays a significant role in aiding patients with movement disorders or disabilities in rehabilitation training and accomplishing activities of daily living [79,97,98,99,100,101], as shown in Figure 6. Basic tasks may pose challenges for individuals with disabilities in the context of daily activities. By integrating EEG technology with external devices, assistive tools can be developed to aid these individuals in accomplishing daily tasks, such as communication assistance or text input. As shown in Figure 6a,b, Marcel F. Hinss et al. utilized a brain EEG acquisition and feedback system to assess the psychological states of individuals, promptly identifying those experiencing suboptimal psychological states among professionals, thereby reducing the occurrence of issues and accidents [79]. Francis R. Willett et al. developed a speech-to-text brain–computer interface system. With the assistance of this system, participants who could no longer speak clearly due to muscle atrophy achieved a 23.8%-word error rate on a vocabulary of 125,000 words, demonstrating effective language communication outcomes [97]. Furthermore, through the control of robotic arms, it is also possible to assist individuals in tasks involving the transportation of goods and the grasping of objects. As in Figure 6c,d. Iason Batzianoulis et al. achieved control of a robotic arm and subsequently the manipulation and transportation of objects using a brain EEG acquisition and feedback system [98]. Similarly, A Bolu Ajiboye et al. proposed a brain EEG control and feedback system that restores limb movements for quadriplegic patients through coordinated electrical stimulation of surrounding muscles and nerves, known as functional electrical stimulation (FES). This enables them to perform simple everyday tasks like grasping a cup [99]. Within rehabilitation training, BCI technology enables individuals with movement impairments to utilize their EEG signals to control external devices such as prosthetics or wheelchairs. Through training, patients can learn to employ specific EEG patterns to execute movements, assisting them in regaining partial limb functionality or improving their daily activities. As shown in Figure 6e,f. Miao et al. collected, analyzed, and classified the EEG signals of stroke patients using a brain EEG acquisition and feedback system. They utilized virtual limbs and functional electrical stimulation (FES) as feedback mechanisms to improve or restore upper limb mobility in stroke patients [100]. Henri Lorach et al. established a brain EEG acquisition and feedback system to restore communication between the brain and spinal cord regions in patients with spinal cord injuries. This, in turn, enabled chronic limb paralysis patients to regain the ability to stand and walk naturally [101]. In the context of monitoring movement recovery, EEG is employed to track changes in the brain activity of individuals undergoing rehabilitation training. This aids rehabilitation professionals in understanding the progress of patient recovery and in adapting rehabilitation plans based on EEG signal variations. Overall, the application of EEG technology in medical rehabilitation and mobility assistance has the potential to significantly enhance the quality of life of individuals with movement disorders or disabilities. Although the application of EEG acquisition systems in motion-assistive systems shows great promise, they also face challenges such as EEG signal noise and interference, complex EEG signal decoding, real-time system performance, and stability. However, with ongoing technological advancements and in-depth research, the application of EEG acquisition systems in motion-assistive systems will continue to advance, providing hope and assistance for individuals requiring motion assistance.

## 4. Data Analysis and Machine Learning in EEG Research

### 4.1. Common Data Analysis Methods

EEG classification algorithms are designed to analyze and categorize EEG data based on specific patterns or features. These algorithms are crucial for understanding and interpreting the brain’s electrical activity [102,103,104,105]. Several commonly used EEG classification algorithms include support vector machines (SVM), K-nearest neighbors (KNN), random forests, convolutional neural networks (CNN), and recurrent neural networks (RNN) [106,107,108,109,110,111,112,113].

### 4.2. Application of Machine Learning in EEG Research

**Figure 7:**
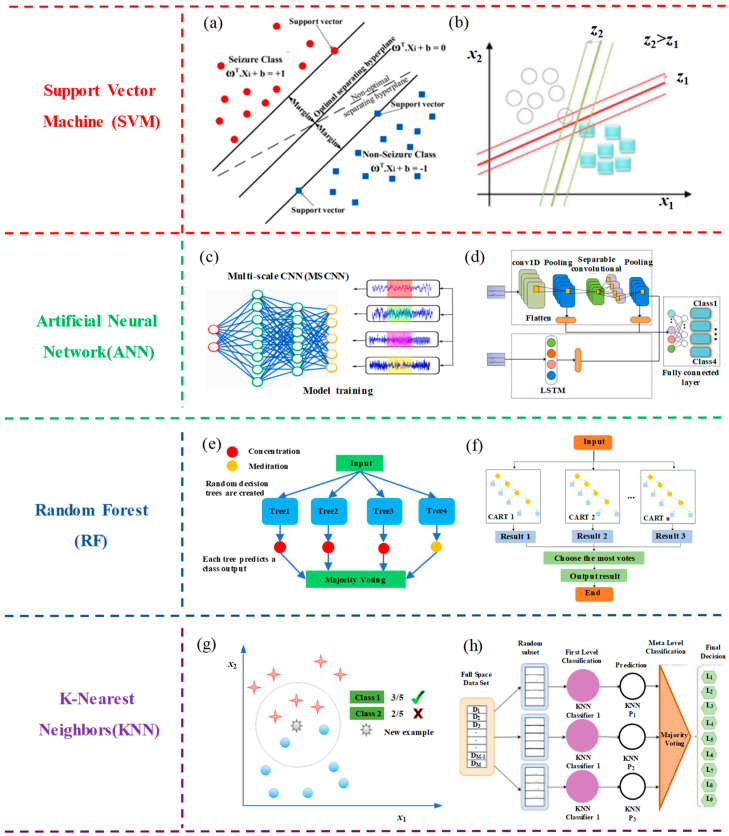
Classification methods for EEG signals. (**a**) Support vector machine (SVM). Reprinted with permission from reference [106]. Copyright 2020, Biomedical Signal Processing and Control. (**b**) Support vector machine (SVM). Reprinted with permission from [107]. Copyright 2020, Journal of Physics: Conference Series. (**c**) Artificial neural network (ANN). Reprinted with permission from [108]. Copyright 2022, Biomedical Signal Processing and Control. (**d**) Artificial neural network (ANN). Reprinted with permission from [109]. Copyright 2022, Biomedical signal processing and control. (**e**) Random forest (RF). Reprinted with permission from [110]. Copyright 2018, Procedia Computer Science. (**f**) Random forest (RF). Reprinted with permission from [111]. Copyright 2022, Journal of Sensors. (**g**) K−nearest neighbors (KNN). Reprinted with permission from [112]. Copyright 2018, Procedia Computer Science. (**h**) K−nearest neighbors (KNN). Reprinted with permission from [113]. Copyright 2023, Journal of Neuroscience Methods.

In the classification of EEG signals, support vector machine (SVM) is employed to identify different brain states or events by extracting features from EEG signals and training models. SVM aims to find the optimal decision boundary that maximizes the margin between different categories. It enhances classification performance through suitable feature extraction, data preprocessing, and cross-validation. SVM plays a crucial role in applications such as neuroscience research and brain–computer interfaces. As shown in Figure 7a, linear discriminant analysis (LDA) was employed to reduce the feature dimensionality [106]. It calculates low-dimensional feature vectors that possess information and discrimination capabilities and is subsequently utilized as input for the SVM classifier. Across 12 experimental studies closely related to clinical applications, this algorithm achieved satisfactory results, with the highest accuracy ranging from 96.25% to 100%. Wijayanto et al. [107] introduced a complexity analysis method for an EEG-based epilepsy detection system using the Higuchi fractal dimension (HFD) for feature extraction. The system integrates an SVM for epilepsy signal classification, as shown in Figure 7b. This approach can be used to predict seizure occurrence, thereby reducing the risk of seizures in patients with epilepsy.

In the classification of EEG signals, the application principles of artificial neural networks (ANNs) encompass several steps, including data preprocessing, feature extraction, the construction of an appropriate neural network architecture, learning mapping relationships from training data, evaluating performance using validation data, and ultimately applying the trained model to classify brain states or events in new data. This approach effectively extracts valuable information from EEG signals, contributing to applications in fields such as neuroscience research and brain–computer interfaces. As shown in Figure 7c, the researchers have introduced an efficient multi-scale convolutional neural network (MS-CNN). This network excels in extracting features from EEG signals across multiple scales for motor imagery (MI) classification. The model achieves an average classification accuracy of 93.74%, surpassing the current state-of-the-art EEG-based MI classification models. The proposed algorithm effectively addresses the limitations of existing CNN-based EEG-MI classification models, resulting in a significant improvement in classification accuracy [108]. As shown in Figure 7d, a neural network feature fusion algorithm is proposed by combining CNN with long short-term memory networks (LSTM). Briefly, spatial features were extracted using a CNN, whereas temporal features were captured using LSTM. Subsequently, all features were combined to enhance classification accuracy. The algorithm achieved an average accuracy of 87.68% [109]. Therefore, this feature fusion neural network effectively enhances the accuracy of motor imagery EEG, offering new insights for feature extraction and classification research on motor imagery-based brain–computer interfaces.

Random forests (RF) is a powerful ensemble learning algorithm widely applied in the classification of EEG signals. This method begins by extracting features from EEG signal data and then constructs multiple decision trees. These decision trees are combined through a voting mechanism to identify different brain states or events. Each decision tree is built based on random samples and features using a bootstrap sampling approach, thereby enhancing model diversity and robustness. Through the voting mechanism, random forests effectively handle complex EEG signal data, improve classification accuracy, and play a crucial role in fields such as neuroscience research, brain–computer interfaces, and clinical applications, while also exhibiting strong generalization capabilities. As shown in Figure 7e, researchers have employed a random forest classifier to construct a brain–computer interface (BCI) model for predicting mental states like concentration and meditation. The analysis and results of this model indicate an accuracy rate of 75% when utilizing the aforementioned approach. This model has been further validated in the internet of things (IoT) domain, showcasing its applicability in home automation applications [110]. As shown in Figure 7f, researchers conducted tests on athletes’ competitive states using a random forest (RF) monitoring model. Experimental results indicate that, compared to the support vector machine (SVM) classification model, the RF model achieves a classification accuracy exceeding 90%. The overall classification accuracy stands at 89.74%, surpassing that of SVM. This study offers valuable insights into monitoring athletes’ competitive states, aiding them in real-time adjustments of their performance levels [111].

The k-nearest neighbors algorithm (k-NN) is employed in the classification of EEG signals by measuring the distances between test data points and the nearest neighbors in the training dataset. The class of a test data point is then determined through a majority voting decision. Key steps in this method include data preprocessing, feature extraction, the selection of an appropriate k-value, distance metrics, classification decisions, and performance evaluation. While k-NN is simple and easy to understand, it performs well in applications with small datasets and high interpretability requirements. It can be used for EEG signal classification to identify different brain states or events. As shown in Figure 7g, binary classification of guilty and innocent classes was carried out using the k-Nearest Neighbors (k-NN) classifier. To validate the deception detection system, each subject underwent 5-fold cross-validation. Among the three parameter sets, the classification accuracy reached 96.7%. This validation underscores the practicality of the classification model in binary classification tasks [112]. As shown in Figure 7h, the proposed multi-channel rhythm-specific features and subspace K-nearest neighbor (SS KNN) method achieved classification accuracies ranging from 93.5% to 99.8% across various emotional states. Compared to previous works, this represents an effective emotion detection approach. Furthermore, the evaluation results indicate that the gamma rhythm, in conjunction with the sub-space KNN, outperforms other EEG rhythms in terms of accuracy [113].

These algorithms have been widely applied across various research and application scenarios, and have achieved notable success. The selection of the most suitable algorithm depends on the specific dataset and task requirements. For smaller datasets and simpler classification tasks, traditional machine learning algorithms such as SVM and RF are often sufficient [114,115,116,117]. However, for large-scale datasets and complex classification tasks, deep-learning algorithms such as CNNs and LSTM may offer more advantages. In practical applications, experimenting with different algorithms and model architectures, often utilizing techniques such as cross-validation, helps to identify optimal algorithmic combination [118,119,120,121].

## 5. Conclusions and Perspectives

Electroencephalography (EEG) data collection technology has evolved significantly, redefining our understanding of the intricate dynamics of the human brain. This technology involves capturing electrical activity produced by neurons using electrodes placed on the scalp. Over time, EEG data collection has transcended conventional neurological research, finding applications across diverse domains and paving the way for transformative advancements. Recent years have witnessed remarkable advances in EEG data collection systems to enhance the precision, efficiency, and practicality of brain research. Innovations in electrode design, signal processing methodologies, and integration with complementary technologies have substantially improved the quality and reliability of acquired data [32,38,41]. High-density EEG systems coupled with 3D electrode configurations have unlocked finer spatial resolutions, providing unprecedented insights into cerebral activity. The emergence of various electrode modalities including dry electrodes and flexible arrays has contributed to increased participant comfort during EEG recordings. Non-invasive alternatives, such as micro-needle and finger-type electrodes, have emerged, minimizing invasiveness and ensuring a more user-centered experience [34,35,36,37].

The future of EEG data collection is promising and multifaceted. Its applications extend beyond traditional research, spanning healthcare, brain–computer interfaces (BCIs), cognitive augmentation, and neuropsychological assessments. The fusion of EEG with artificial intelligence, machine learning, and advanced data analytics is poised to reveal novel dimensions of brain function, leading to more precise diagnostics and tailored interventions for neurological disorders [106,107,108,109,110,111,112,113]. Furthermore, the synergy of EEG technology with other biometric data, such as eye tracking and heart rate variability, promises a holistic comprehension of cognitive and emotional states. This multidimensional approach holds the potential to revolutionize areas such as mental health diagnosis, neurofeedback, and brain-controlled devices. Nonetheless, challenges remain, including the need for enhanced noise mitigation techniques, standardization of data collection protocols, and addressing current limitations of EEGs in capturing deep-seated brain structures. Persistent research efforts and interdisciplinary collaborations will be pivotal in overcoming these challenges and refining EEG data collection technology [64].

In summary, EEG data collection technology has reshaped neuroscience and several other fields, empowering researchers to delve into the enigmatic realm of the human mind. As advancements continue and diverse fields converge, the horizon for EEG technology appears rich with possibilities and promising deeper insights into the complexities of cerebral function.

## Data Availability

Not applicable.
